# Synthesis of Graphene-like Materials from Acetylene Black, Activated Carbon, and Ketjenblack via Separated Microwave Electric and Magnetic Field Heating

**DOI:** 10.3390/ma16103723

**Published:** 2023-05-14

**Authors:** Takeshi Miyata, Syun Gohda, Akio Oshita, Hironobu Ono, Keiichiro Kashimura

**Affiliations:** 1 Faculty of Engineering, Chubu University, 1200 Matsumoto-cho, Kasugai 487-8501, Aichi, Japan; 2 Nippon Shokubai Co., Ltd., Nishi-Otabicho, Suita 564-0034, Osaka, Japan

**Keywords:** acetylene black, activated carbon, Ketjenblack, graphene, microwave heating

## Abstract

Acetylene black, activated carbon, and Ketjenblack were subjected to microwave heating up to 1000 °C under N_2_ atmosphere to rapidly convert them into graphene-like materials. Few carbon materials exhibit a favorable increase in the intensity of the G’ band with increasing temperature. Upon electric field heating of acetylene black to 1000 °C, the observed relative intensity ratios of D and G bands (or G’ and G band) were equivalent to those of reduced graphene oxide heated under identical conditions. In addition, microwave irradiation under different conditions, i.e., electric field or magnetic field heating, produced graphene of qualities different from those of the same carbon material conventionally treated at the same temperature. We propose that this difference arises from the different mesoscale temperature gradients. The conversion of inexpensive acetylene black and Ketjenblack into graphene-like materials within 2 min of microwave heating is a major achievement toward low-cost mass synthesis of graphene.

## 1. Introduction

In recent years, carbon has been actively studied as a strong material with excellent thermal and electrical properties. Studies on carbon materials, such as fullerenes, carbon nanotubes, carbon nanobrushes, carbon microcoils, and graphite intercalation compounds, have been reported for decades [1,2,3,4,5]. Among these carbon materials, graphene attracts significant attention and has potential applications in fields such as electronics, composite materials, medicine, and textiles, owing to its excellent mechanical strength, thermal/electrical conductivity, and thin-film properties [6,7,8,9,10].

Since its discovery in 2004, researchers have developed various methods for synthesizing graphene. Currently, graphene is synthesized via methods such as graphite exfoliation, chemical vapor deposition (CVD), epitaxial growth, and the heat reduction of graphene oxide. Graphite exfoliation, CVD, and epitaxial growth can be used to synthesize graphene with high carrier mobility. However, these methods have drawbacks, such as difficulty in mass synthesis, high cost of raw materials, and pollution of the environment during synthesis [11,12,13]. The thermal reduction of graphene oxide is excellent for mass productivity; however, the quality of the synthesized graphene is insufficient for practical use. Furthermore, the handling of graphene oxide as a raw material is challenging, which is a limitation of the heat reduction method.

In this context, a heat reduction method that converts carbon materials and exhaust gas into graphene is both an ecofriendly and low-cost means to produce graphene in large quantities. For example, Li et al. developed a method to convert petroleum-derived asphalt into graphene by performing pulse arc discharge in water [14]. Chakrabarti et al. reported that burning Mg in a CO_2_ atmosphere reduces CO_2_ and deposits graphene on the oxidized magnesium surface [15]. Additionally, Akhavan et al. used graphited wood, fruit waste, and soot waste from diesel vehicles to synthesize graphene oxide, which was then reduced to obtain graphene [16]. Loung et al. showed that gram-scale graphene can be generated in about 1 s by Joule heating of cheap carbon sources such as waste food. Their products are more than 99% carbon pure, do not require refining, and have an electrical energy cost of around 7.2 kJ/g [17]. Furthermore, Islam et al. used plasma-assisted thermal shock to exfoliate graphene from graphite at a rate as high as 48 g/h. They report high reproducibility of the technique, with the graphene retaining the same quality across different batches [18].

In this study, we focused on microwave (MW) irradiation conditions (microwave component and heating temperature) to repair defects and increase the π bonding in carbon materials. MW irradiation has been reported to rapidly convert reduced graphene oxide (rGO) to graphene, repair defects, and remove impurities [19,20,21,22]. Previous studies have reported that using special microwaves with enhanced magnetic fields can improve this defect-repair and impurity-removal effect [23]. We irradiated three typical carbon materials (acetylene black, activated carbon, and Ketjenblack) by use of a single mode waveguide applicator in which the maximum electric and magnetic fields are well separated and then investigated its effects on defects and π bonding. MW irradiation conditions (electric field or magnetic field heating) and temperature (700–1000 °C) were used as parameters to evaluate the defects and impurities in the obtained carbon material.

## 2. Materials and Methods

**Figure 1:**
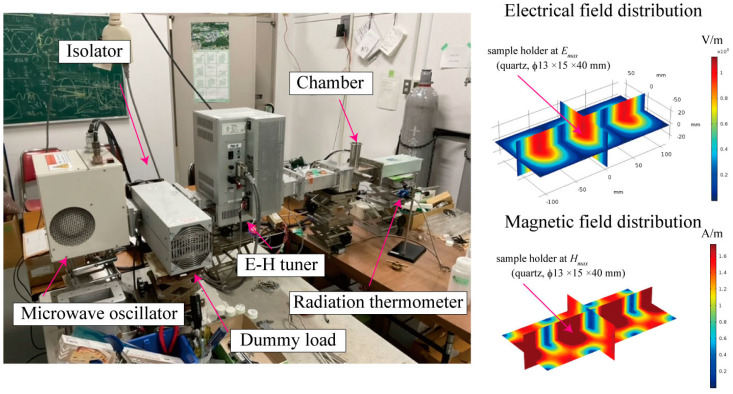
A photograph showing the single−mode heating device and the calculated electromagnetic field intensity distribution inside the device during microwave irradiation. Here, the microwave power is assumed to be 1 W.

Acetylene black (manufactured by Denka, Tokyo, Japan), activated carbon (manufactured by Kuraray and Osaka Gas Chemical Co., Ltd., Osaka, Japan), and Ketjenblack (manufactured by Lion, Tokyo, Japan) were used as the screening test samples. Activated carbon raw materials were selected from samples made by Osaka Gas Co., Ltd., Osaka, Japan, and Kuraray Co., Ltd., Tokyo, Japan, which are derived from wood. Activated carbon made by Osaka Gas (Activated carbon (O)) is activated with zinc chloride. Activated carbon (O) is derived from biomass and is characterized by its many fine structures. The activated carbon from Kuraray (Activated carbon (K)) is said to be derived from coal. These samples were exposed to pure electric or magnetic fields at 2.45 GHz MW irradiation using a cavity resonator. The heating system consisted of a standard TE102 waveguide resonator (109.2 mm × 54.6 mm × 149.3 mm) with a magnetron oscillator (MPS-17A, Nisshin Engineering Corp., Tokyo, Japan; frequency: 2.455 GHz ± 20 MHz; output power: 1.7 kW) electric–magnetic (E–H) tuner, plunger, and dummy load, as shown in Figure 1. The MWs were coupled into the TE102 waveguide resonator. The iris consisted of a 28 mm slit parallel to the direction of the electric field. The plunger was placed at the end of the waveguide. This system enabled the spatial separation of the electric and magnetic fields for the MW [24,25]. The samples (0.2 g) were inserted into the fused-quartz sample holder (Φ13 mm ×15 mm × 40 mm), which was placed at an electric field node (denoted by *E_max_*, where the magnetic field is zero) or at a magnetic field node (*H_max_*, where the electric field is zero). The temperatures of the samples were monitored using a radiation thermometer (FTZ6-R220-5S22, Japan Sensor Corp., Tokyo, Japan). To prevent the carbon materials from burning, 99.99% pure (4N) N_2_ gas flowed at a rate of 0.6 L/min. The cavity was purged for 3 min before heat treatment. To confirm the superiority of microwave heating, the carbon materials were compared after microwave and electric furnace heating. In the electric furnace heating experiment, the sample was weighed using a 0.20 g precision balance (A & D Co., Ltd., Tokyo, Japan: GH-202, 0.2 ± 0.01 g), loaded into an alumina boat, and heated in an electric furnace (Motoyama Co., Ltd., Osaka, Japan: NLT-2035-SP). Similar to microwave heating, N_2_ gas also flowed at a rate of 0.6 L/min. After reaching the final temperature, which was held for 2 min, the heating experiment was terminated.

The sample was heated to a predetermined temperature (700–1000 °C) at *E_max_* or *H_max_* and maintained at that temperature for 2 min. After MW heating, the samples were analyzed using Raman spectroscopy (JASCO. Co., Ltd., Tokyo, Japan, NRS-3100) with an excitation wavelength of 532 nm, a 20× objective lens, exposure time of 1 s, and 32 averaged integrations. The three peaks that were considered are the D band (1300–1400 cm^–1^), G band (1550–1610 cm^–1^), and G’ band (2650–2750 cm^–1^). A decrease in the D band intensity and an increase in the G and G’ band intensities were regarded as indicators for the quality of defect repair and graphene formation. Surface impurities of carbon materials were investigated using X-ray photoelectron spectroscopy (XPS; Shimazu. Co. Ltd., Kyoto, Japan, AXIS-NOVA). Al-Kα was selected as the source and pass energy of 40 eV was adopted. The neutralization gun was on during the XPS measurement. The Thermera-NIR2 system (Mitsui Photonics Ltd., Tokyo, Japan) was employed to measure the mesoscale temperature at approximately 900 °C using two wavelengths (800 and 975 nm). A two-dimensional, two-color thermometer uses two wavelengths to calculate the temperature from the difference. Therefore, the influence of the thickness of quartz and dirt on the window material can be canceled. This system enabled the temperature measurement of a particle in the mixture [26] with a spatial resolution of 2.2 µm/pixel.

To obtain reproducibility, all measurements were performed in triplicate, including the heating step.

## 3. Results

Raman spectroscopy was used to assess the structural defects and domain sizes of the samples. The peak at approximately 1300 cm^–1^ is the D band, which represents structural defects in graphene. Low intensity of the D band peak indicates fewer graphene defects [27,28]. The peak at 1600 cm^–1^ is the G band, which arises from the graphene domains. The sharper the G band peak, the larger the graphene domain size. The decrease in the D band and sharpening of the G band indicates an increase in π bonding of the carbon material, and that their structure has become closer to that of graphene [27,28,29]. The G’ band at 2700 cm^–1^ arises from the p-electron scattering of graphene and can be used to determine the number of layers present; sharper peaks indicate less p-electron scattering, and thus fewer layers [29,30,31]. The sharp peak around 2830 cm^–1^ is from instrumental noise, while the peak at 2950 cm^–1^ is derived from defects and is called the D + G band.

**Figure 2:**
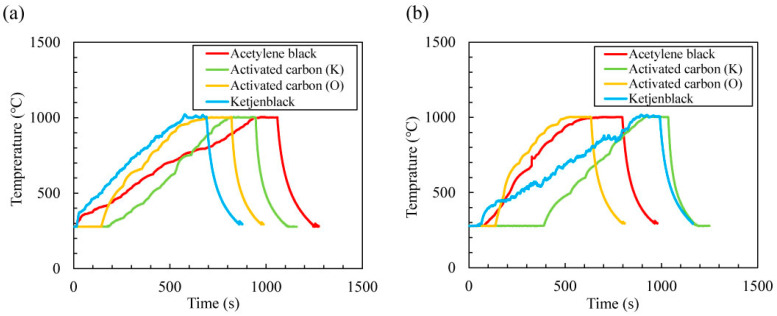
(**a**) Temperature change when 0.2 g carbon materials were heated by microwave electric and (**b**) magnetic fields (holding time: 2 min, holding temperature: 1000 °C), where 99.99% N_2_ gas flowed into the cavity (flow rate: 0.6 L/min).

All carbon materials were well heated using microwave electric and magnetic fields. Figure 2 shows the temperature change over time when acetylene black, activated carbon (K), activated carbon (O), and Ketjenblack were heated using (a) pure microwave electric or (b) magnetic fields, where the temperature was maintained at 1000 °C for 2 min. The microwave electric field outputs required to increase the temperature to 1000 °C for acetylene black, activated carbon (K), activated carbon (O), and Ketjenblack were 290–420, 155–220, 175–210, and 240–275 W, respectively. Furthermore, the microwave magnetic field outputs required to reach 1000 °C for acetylene black, activated carbon (K), activated carbon (O), and Ketjenblack were 210–320, 210–235, 170–260, and 300–405 W, respectively. Moreover, compared with a pure microwave magnetic field, a pure microwave electric field can heat activated carbon (K), activated carbon (O), and Ketjenblack more quickly. The microwave output power described was at steady temperature for three measurements of each sample, and detailed profiles of the temperature and input/output microwave powers are shown in Supplementary Figure S1.

The difference in heating rate of carbon materials is influenced by the difference in their electrical conductivities. In a microwave electric field, the microwave absorption of a material is proportional to the product of the imaginary part of the permittivity and E^2^. The imaginary part of the permittivity consists of the contribution due to the polarization of the particle and that due to conductivity. Because these are larger owing to the crystallite size of the carbon material, there is a difference in the heating rate. In a microwave magnetic field, the microwave absorption of a material is proportional to the product of the imaginary part of the permeability and H^2^. In carbon materials, which are weakly magnetic, the porcelain losses are assumed to consist of induced currents. Therefore, the difference in conductivity of the carbon material would considerably affect the heating rate. Considering results from previous research [32], the difference in particle shape and packing rate may have also affected the heating rate.

**Figure 3:**
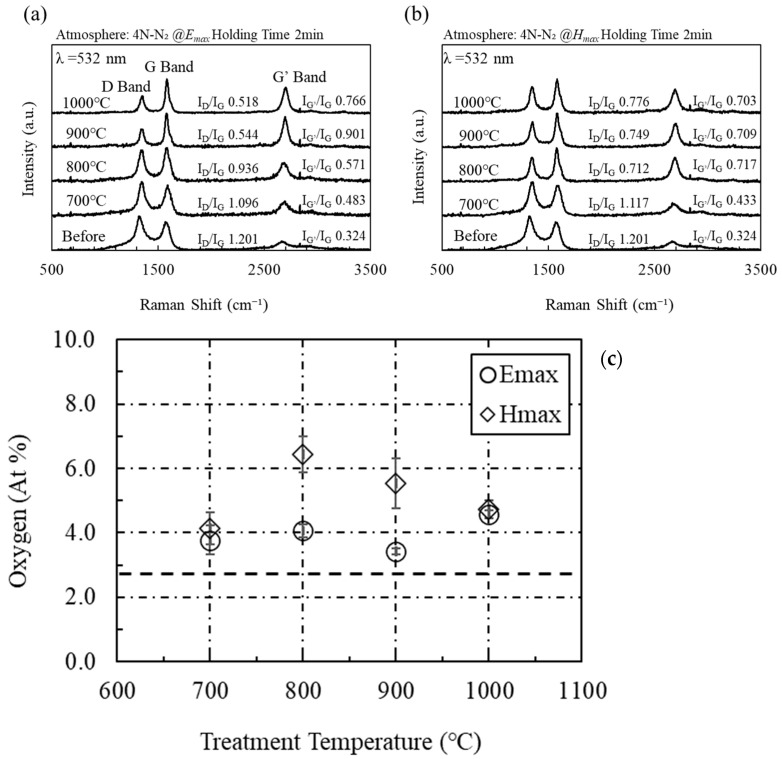
(**a**) Raman spectra of acetylene black heated by the microwave electric field and (**b**) the magnetic field, and (**c**) the average amount of oxygen as measured by XPS on the surface of acetylene black after heating with microwave electric and magnetic fields (trial number: 3). Here, the dotted line shows the amount of oxygen on the surface of acetylene black before microwave irradiation.

Compared with conventional heating (CH), microwave electric field heating of acetylene black is extremely effective for repairing defects and increasing π bonding owing to the decrease in I_D_/I_G_ ratio and increase in I_G’_/I_G_ ratio (Supplementary Figure S2). Figure 3 shows the Raman spectra from acetylene black heated using microwave electric and magnetic fields. The sample heated with the microwave electric field shows lower D band and higher G band intensities than those heated with the microwave magnetic field and electric furnace. This indicates that the defects in acetylene black are repaired via microwave electric field heating [27,28]. The microwave magnetic field increases the intensity and decreases the width of the G’ band of acetylene black. The sharper the G band peak, the larger the graphene domain size [27,29]. The quality of the graphene resulting from both heating methods improves with increasing temperature.

Figure 3c shows the amount of oxygen as measured by XPS on the surface of acetylene black after heating with microwave electric and magnetic fields (Shimadzu Kratos, Manchester, UK, AXIS-NOVA; 100-W Al-Kα X-ray source with a pass energy of 40 eV and neutralization gun turned on). Here, the error bar was determined by using the standard deviation (number of trials: 3). The oxygen content on the surface of acetylene black before microwave irradiation is 2.9 at%, whereas a value of 4.0 at% or more is observed after microwave irradiation. In previous work, we irradiated rGO with microwaves to remove its impurities, and we confirmed that microwave irradiation reduced surface oxygen [23]. Microwave heating is effective in removing impurities on the rGO surface but not in removing impurities on the surface of acetylene black.

**Figure 4:**
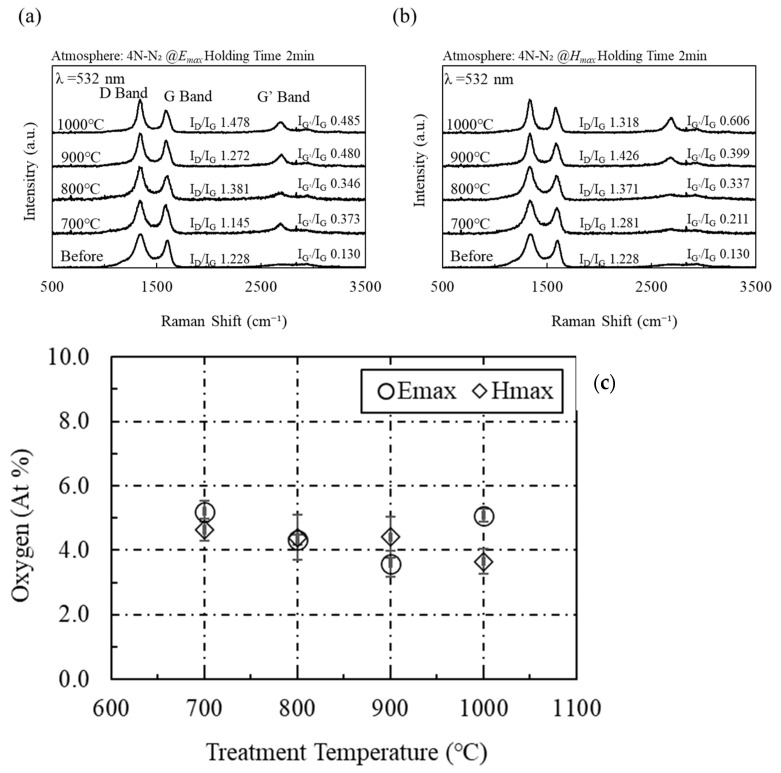
(**a**) Raman spectra of activated carbon (K) heated by the microwave electric field and (**b**) the magnetic field, and (**c**) the average amount of oxygen as measured by XPS on the surface of activated carbon (K) after heating with microwave electric and magnetic fields (trial number: 3).

**Figure 5:**
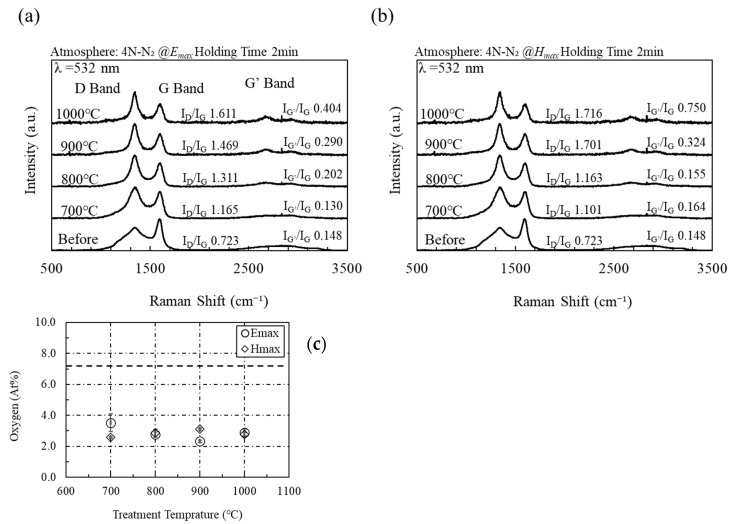
(**a**) Raman spectra of activated carbon (O) heated by the microwave electric field and (**b**) the magnetic field, and (**c**) the average amount of oxygen as measured by XPS on the surface of activated carbon (O) after heating with microwave electric and magnetic fields (trial number: 3).

Although microwaves were effective in graphenization and impurity removal from rGO, microwave heating of activated carbon is not effective for repairing defects. Figure 4 shows the Raman spectra from activated carbon (K) heated using microwave electric and magnetic fields. Unlike those of acetylene black, the Raman spectra of activated carbon (K) show that microwaves do not affect the D and G bands. This indicates that the microwave electric and magnetic fields are not effective in repairing defects in activated carbon (K). In addition, the G’ band expands when irradiated with the microwave electric or magnetic field. Thus, microwaves are effective for the crystallization of activated carbon (K). Figure 4c shows the amount of oxygen as measured by XPS on the surface of activated carbon (K) after heating with the microwave electric and magnetic fields. The oxygen content on the surface of activated carbon (K) before microwave irradiation is 7.3 at%, whereas a value of approximately 4 at% after microwave irradiation (*H_max_*) is observed. Although the microwaves were not effective in removing impurities from the rGO surface, the microwave electromagnetic field is effective in removing impurities from the activated carbon (K) surface. As shown in Figure 5, an identical trend is observed in activated carbon (O) manufactured by different manufacturers. Thus, the experimental results show that microwaves are effective only for the crystallization of activated carbon and not for defect repair or surface impurity removal.

**Figure 6:**
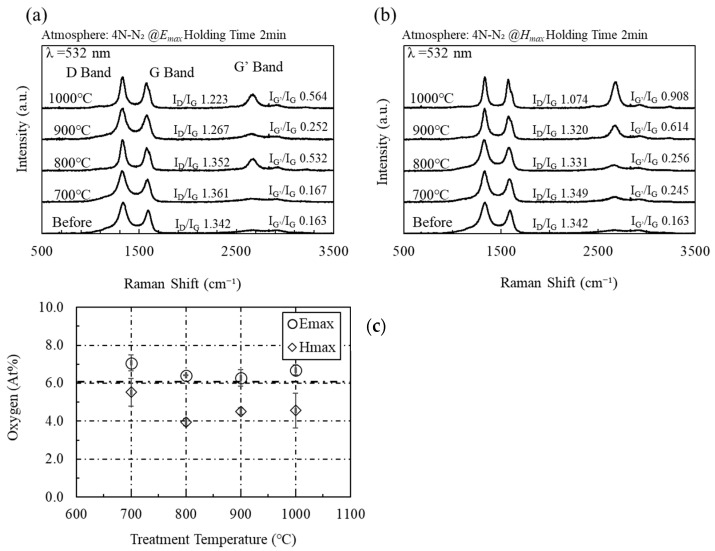
(**a**) Raman spectra of Ketjenblack heated by the microwave electric field and (**b**) the magnetic field, and (**c**) the average amount of oxygen as measured by XPS on the surface of Ketjenblack after heating with microwave electric and magnetic fields (trial number: 3).

The microwave magnetic field is effective in increasing the π bonding of Ketjenblack and in the removal of surface impurities. Figure 6 shows the Raman spectra from Ketjenblack heated using microwave electric and magnetic fields. The 2D and G bands of Ketjenblack after microwave electric field heating exhibit slight changes in shape but show no signs of increasing π bonding and defect repair. Contrastingly, the microwave magnetic field narrows the G’ band of Ketjenblack. The G’ band becomes sharper with increasing temperature, and the band sharpness is higher under microwave magnetic field treatment than under electric field heating. Figure 6c shows the amount of oxygen as measured by XPS on the surface of Ketjenblack after heating via microwave electric and magnetic fields. According to the XPS results, the Ketjenblack surface contains 6 at% oxygen before microwave heating, and the surface heated using the microwave magnetic field contains approximately 4 at% oxygen, except for products heated at 700 °C, whereas Ketjenblack heated using the microwave electric field contains at least 6 at% oxygen on its surface. Thus, the microwave magnetic field is effective in increasing π-bonds and in removing oxygen impurities from the surface of Ketjenblack.

**Figure 7:**
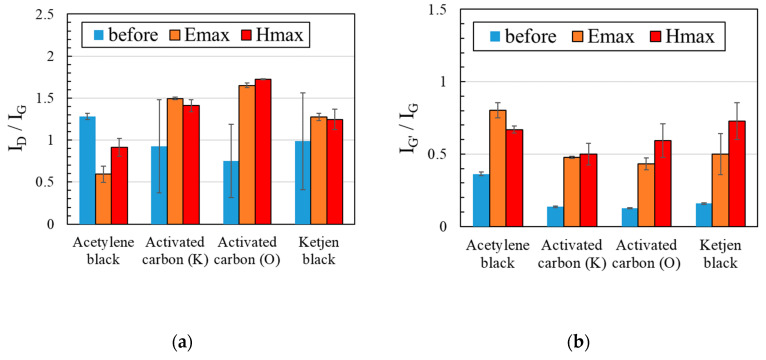
Comparison of the (**a**) Raman spectra ID/IG and (**b**) IG’/IG ratios of carbon materials heated at 1000 °C for 2 min. Here, the experiment was performed three times and the average values are plotted as a graph.

**Figure 8:**
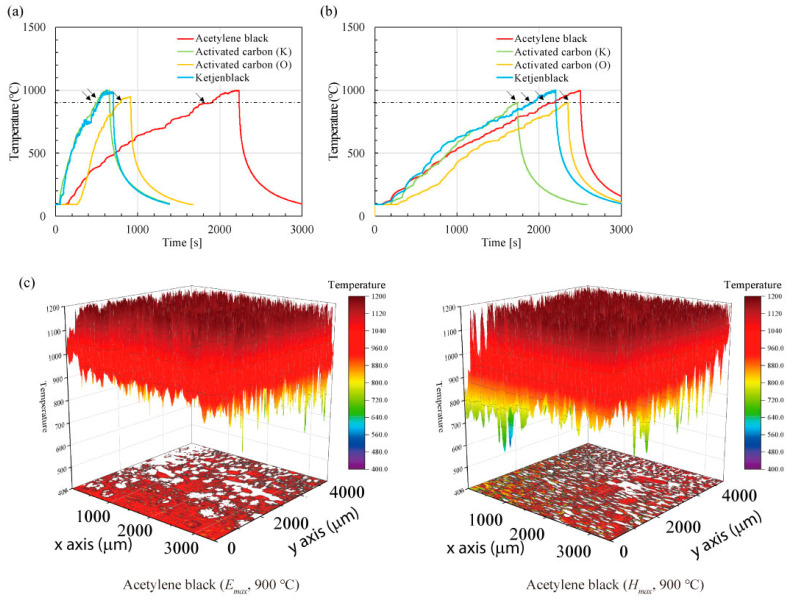

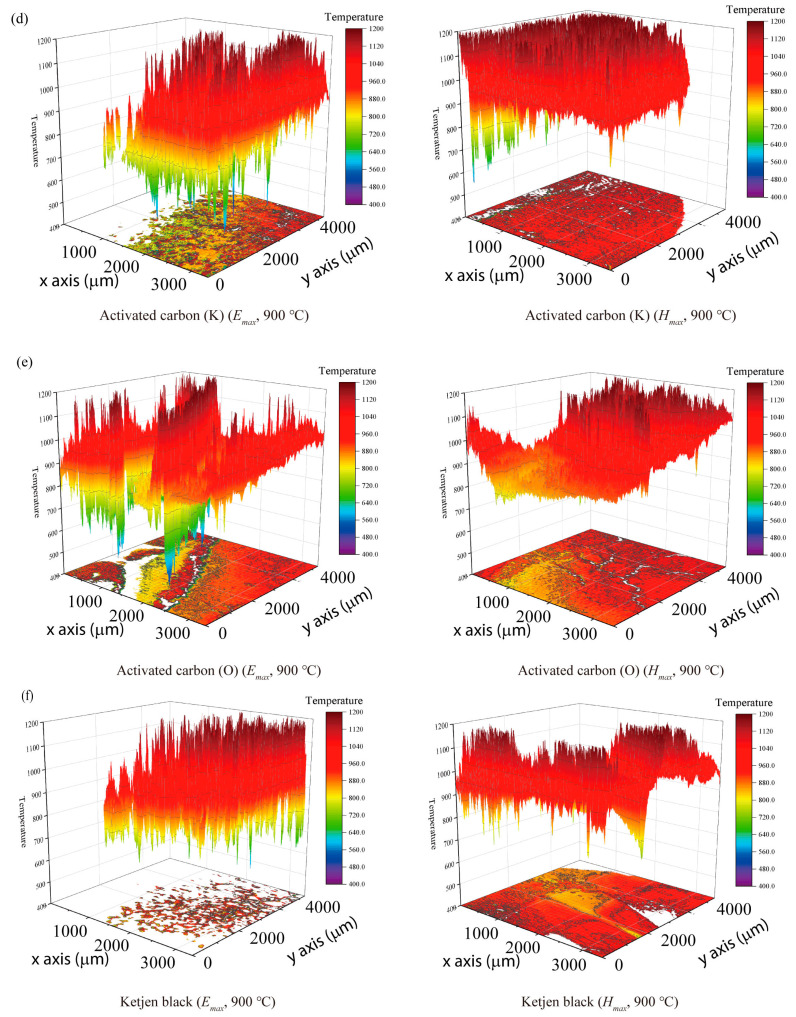
(**a**,**b**) Temperature vs. time plots for carbon materials heated by the microwave electric field or the magnetic field. The temperature was measured using a monochromatic thermometer. A narrow temperature distribution (2.8 μm/pixel) is shown for each carbon material (**c**–**f**). The white areas correspond to areas outside the measurement range.

Among the carbon materials and heating conditions considered, the electric field heating of acetylene black is the most promising candidate for the graphene precursor, as shown in Figure 7. Here, the experiment was performed three times and the average values are plotted. The high-speed and selective heating, which are the characteristics of microwave heating, could have led to the effective defect repair and crystallization of acetylene black. Figure 8 shows changes through time in the temperature of carbon materials heated using microwave electric or magnetic fields, recorded using a monochromatic thermometer, and the narrow temperature distribution (2.8 μm/pixel) of each carbon material. Although all carbon materials are at 900 °C at the time indicated by the arrows in Figure 8a,b, the temperature distribution differs for each carbon material in the order of μm. In the electric field heating of acetylene black, small white and red dots are observed in the contour diagram shown in Figure 8. This indicates that the temperature gradient is significantly large when heating these carbon materials. We claim that this large temperature gradient contributes to the defect repair.

In all carbon materials, MW is superior to CH in producing graphene-like crystal structures. In CH, graphene formation is challenging, and the G band shifts to high wavenumbers (1600 cm^–1^), which may be because of an increase in the D’ band component derived from defects. Furthermore, the G band of the MW-irradiated sample approaches that of graphene (1580 cm^–1^), suggesting formation of a graphene-like material (Supplementary Table S1). In addition, the top G band peak of the sample treated with magnetic field heating is closer to 1580 cm^–1^ than that treated with electric field heating (according to the 1000 °C data for ACT-K, ACT-O, and KTB).

When viewed macroscopically, there could be two reasons for the homogeneous magnetic field heating. One is that the magnetic field heating does not excite the plasma, so it only heats the material. Thin plasma may have been generated during electric field heating. Second, the magnetic field penetrates deep into the material and homogeneous heating is possible. There should be free electrons on the surface of the hot carbon material; however, these electrons reflect the electric field. As the magnetic field is not bound by these free electrons, it can penetrate deeper than the electric field.

Defect repair can be explained by considering local thermal equilibrium during microwave heating. From a thermodynamic point of view, the reaction 2CO ↔ C + CO_2_ significantly contributes to the defect repair. When the partial pressure of CO_2_ is low, this reaction proceeds to the right at low temperatures and to the left at high temperatures [33,34]. As microwaves cannot heat the gas, the CO gas emitted from the carbon material is colder than that in an electric furnace, i.e., the cold CO gas attaches to the defects and repairs the crystals of the carbon material. However, microwaves can heat carbon materials well. A carbon material efficiently emits CO gas according to the Boudouard curve (Supplementary Figure S2). Therefore, acetylene black, which has both these characteristics, can achieve good defect repair and crystallization. However, for other carbon materials, the magnitude is large, and the gradient is moderate, although a temperature gradient peculiar to microwave heating is observed. In addition, considering that a normal radiation thermometer indicates 900 °C, the white area indicated by the two-dimensional, two-color thermometer is presumed to be a low temperature area. Virtually no region with low temperature is observed. Although crystallization is observed with activated carbon, it is presumed that the defect repair is insufficient. Repair of certain defects has been observed in activated carbon; however, the defect repair is less effective compared to that in acetylene black and Ketjenblack. This is attributable to the formation of an atmosphere that inhibits carbon deposition on the surface of activated carbon.

## 4. Conclusions

A cavity resonator was employed to separate pure electric and magnetic fields, and acetylene black, activated carbon, and Ketjenblack were heated using the strong electric or magnetic microwave fields. The defect repair efficiency and surface oxygen concentration of the acetylene black, activated carbon, and Ketjenblack after treatments were measured using Raman spectroscopy and XPS, revealing the following results:

(1) In acetylene black, activated carbon, and Ketjenblack, microwave heating was more effective for defect repair and crystallization than electric furnace heating. Defect repair and increased π bonding were observed as the microwave irradiation temperature increased.
(2) The number of oxygen atoms adhering to the activated carbon surface was reduced during microwave heating. Defect repair by microwave heating in activated carbon is inferior to that in acetylene black.
(3) Microwave-heated acetylene black, activated carbon, and Ketjenblack had a region where the temperature is approximately 200 °C higher than those of the surrounding regions, and the magnitude tended to differ depending on the electric and magnetic fields.
(4) Acetylene black is a promising raw material for graphene synthesis via microwave heating.

The reason for the more effective defect repair using microwave heating than using electric furnace heating is thought to be from the temperature difference between the cold CO gas and hot carbon particles. Moreover, with respect to mesoscale thermodynamics, microwave heating is more effective for defect repair than electric furnaces because the heating region is smaller (<2.8 μm). Using a catalyst and gas atmosphere, this technology can potentially convert inexpensive carbon materials, such as acetylene black, activated carbon, and Ketjenblack, into graphene precursors.

## Data Availability

Not applicable.

## Supplementary Materials

The following supporting information can be downloaded at: https://www.mdpi.com/article/10.3390/ma16103723/s1. Table S1: Selected peak shifts from the Raman spectrum analysis of each carbon material; Figure S1: Temporal profiles of the temperature and microwave input/reflected power of each carbon material; Figure S2: Temperature dependence of the Gibbs energy change of reaction: 2CO⇒C+CO_2_ (where P_CO_ = 4.0 × 10^–4^ atm, P_CO2_ = 4.0 × 10^–8^ atm). Low-temperature CO gas is deposited as carbon. Where, ΔG indicates the Gibbs free energy change.
