# Supplementary material for: Synthesis of Graphene-like Materials from Acetylene Black, Activated Carbon, and Ketjenblack via Separated Microwave Electric and Magnetic Field Heating
Source: Materials (Basel). 2023 May 14;16(10):3723. doi: 10.3390/ma16103723 (PMC10223618; doi:10.3390/ma16103723)
Supplement: Supplementary file 1 [file materials-16-03723-s001.zip › materials-2321106-supplementary.pdf]

Supplementary Material

Synthesis of Graphene-Like Materials from Acetylene Black, Activated Carbon, and Ketjenblack via Separated Microwave Electric and Magnetic Field Heating

Takeshi Miyata [1,*], Syun Gohda [2], Akio Oshita [1], Hironobu Ono [2] and Keiichiro Kashimura [1,*]

[1] Faculty of Engineering, Chubu University, 1200 Matsumoto-cho, Kasugai 487-8501, Aichi, Japan
[2] Nippon Shokubai Co., Ltd., Nishi-Otabicho, Suita 564-0034, Osaka, Japan
* Correspondence: tk20016-5404@sti.chubu.ac.jp (T.M.); kashimura@isc.chubu.ac.jp (K.K.); Tel.: +81-568-51-8420; Fax: +81-568-51-8421

Table S1. Selected peak shifts from the Raman spectrum analysis of each carbon material.

**ASB**	D/ cm^{-1}	G/ cm^{-1}	G'/ cm^{-1}
Before	1325	1575	2665
CH	1353	1607	2680
E700	1345	1590	2695
E800	1345	1583	2690
E900	1350	1585	2697
E1000	1353	1583	2702
H700	1345	1585	2690
H800	1347	1580	2695
H900	1352	1583	2700
H1000	1347	1583	2695

**ACT(K)**	D/ cm^{-1}	G/ cm^{-1}	G'/ cm^{-1}
Before	1345	1605 broad	
CH	1362	1615	2685
E700	1343	1588	2683
E800	1345	1598	2685
E900	1345	1592	2688
E1000	1340	1588	2685
H700	1337	1592	2685
H800	1335	1592	2685
H900	1340	1590	2690
H1000	1338	1584	2693

**ACT(O)**	D/ cm^{-1}	G/ cm^{-1}	G'/ cm^{-1}
Before	1350	1592 broad	
CH	1365	1615	2680
E700	1342	1598	2665
E800	1343	1599	2670
E900	1340	1600	2670
E1000	1347	1600	2675
H700	1345	1598	2680
H800	1348	1595	2680
H900	1340	1595	2675
H1000	1340	1588	2680

**KTB**	D/ cm^{-1}	G/ cm^{-1}	G'/ cm^{-1}
Before	1333	1592	2670
CH	1352	1607	2670
E700	1342	1595	2680
E800	1340	1598	2675
E900	1340	1598	2670
E1000	1343	1593	2670
H700	1345	1597	2680
H800	1340	1595	2675
H900	1336	1595	2675
H1000	1339	1585	2675

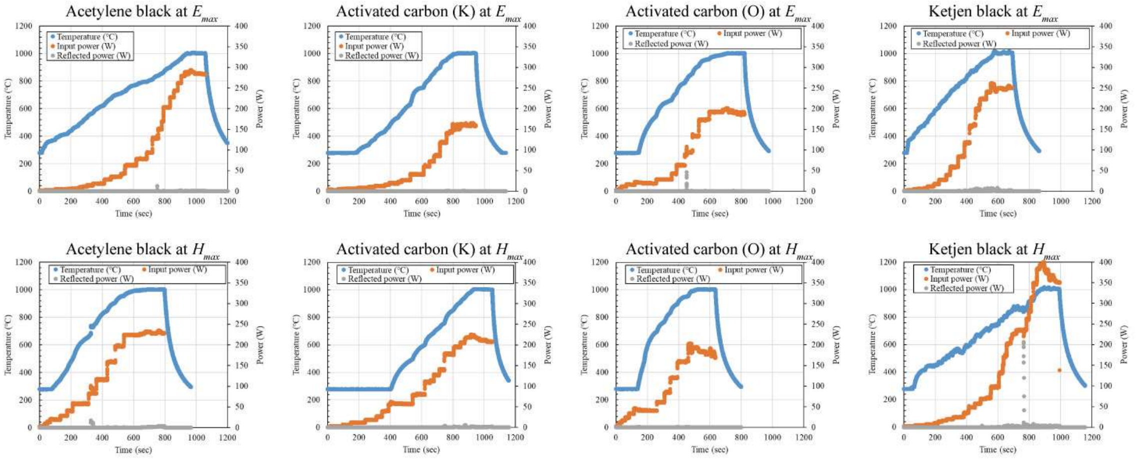

Figure S1. Temporal profiles of the temperature and microwave input/reflected power of each carbon material.

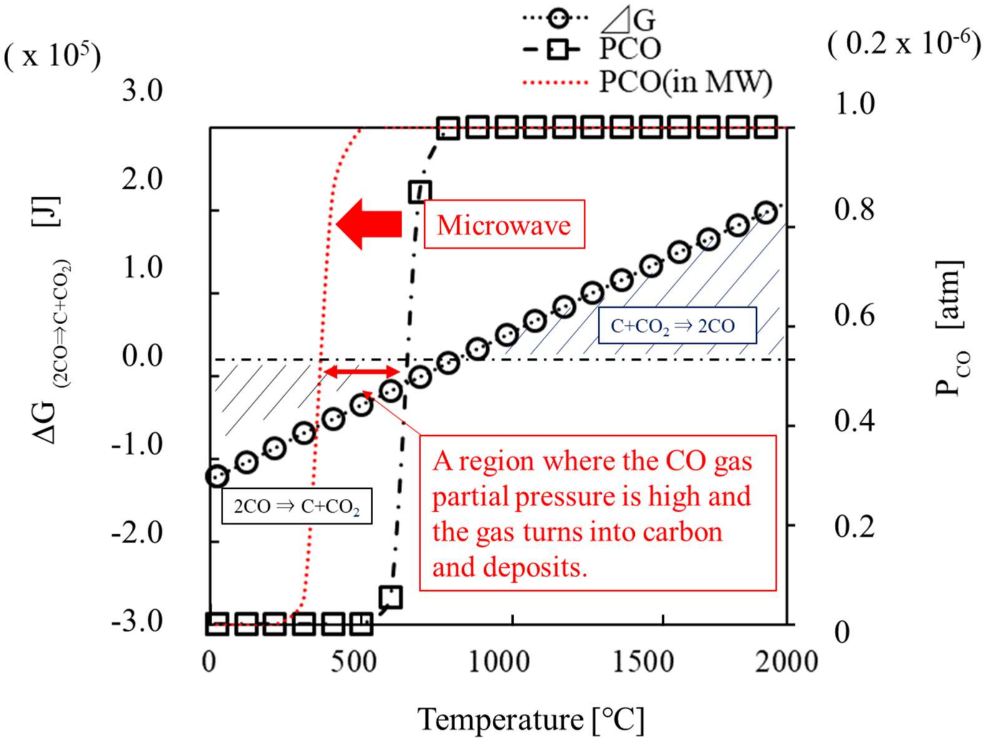

Figure S2. Temperature dependence of the Gibbs energy change of reaction: $2CO \Rightarrow C+CO_2$ (where P_{CO}=4.0 ×10^{-4} atm, P_{CO2}=4.0 ×10^{-8} atm). Low-temperature CO gas is deposited as carbon. Where, ΔG indicates the Gibbs free energy change.
